# Nomogram Model Based on Clinical Risk Factors and Heart Rate Variability for Predicting All-Cause Mortality in Stage 5 CKD Patients

**DOI:** 10.3389/fgene.2022.872920

**Published:** 2022-05-16

**Authors:** Xueyan Gao, Jing Wang, Hui Huang, Xiaoxue Ye, Ying Cui, Wenkai Ren, Fangyan Xu, Hanyang Qian, Zhanhui Gao, Ming Zeng, Guang Yang, Yaoyu Huang, Shaowen Tang, Changying Xing, Huiting Wan, Lina Zhang, Huimin Chen, Yao Jiang, Jing Zhang, Yujie Xiao, Anning Bian, Fan Li, Yongyue Wei, Ningning Wang

**Affiliations:** ^1^ Department of Nephrology, The First Affiliated Hospital of Nanjing Medical University, Jiangsu Province Hospital, Nanjing, China; ^2^ Department of General Medicine, Geriatric Hospital of Nanjing Medical University, Nanjing, China; ^3^ Department of Epidemiology and Biostatistics, School of Public Health, Nanjing Medical University, Nanjing, China; ^4^ Department of Nephrology, Northern Jiangsu People’s Hospital Affiliated to Yangzhou University, Yangzhou, China; ^5^ Department of Nephrology, Affiliated Dongyang Hospital of Wenzhou Medical University, Dongyang, China; ^6^ Department of Nephrology, The Second Affiliated Hospital of Jiaxing University, Jiaxing, China; ^7^ Department of Nephrology, BenQ Medical Center, The Affiliated BenQ Hospital of Nanjing Medical University, Nanjing, China; ^8^ Department of Nephrology, Henan Provincial Key Laboratory of Kidney Disease and Immunology, Henan Provincial People’s Hospital, People’s Hospital of Zhengzhou University, Zhengzhou, China; ^9^ Department of Nephrology, Taizhou People’s Hospital, Taizhou, China; ^10^ Department of Nephrology, The Third People’s Hospital of Jingdezhen, Jingdezhen, China; ^11^ China International Cooperation Center for Environment and Human Health, Nanjing Medical University, Nanjing, China

**Keywords:** chronic kidney disease, circadian rhythm, heart rate, heart rate variability, all-cause mortality, nomogram model

## Abstract

**Background:** Heart rate variability (HRV), reflecting circadian rhythm of heart rate, is reported to be associated with clinical outcomes in stage 5 chronic kidney disease (CKD5) patients. Whether CKD related factors combined with HRV can improve the predictive ability for their death remains uncertain. Here we evaluated the prognosis value of nomogram model based on HRV and clinical risk factors for all-cause mortality in CKD5 patients.

**Methods:** CKD5 patients were enrolled from multicenter between 2011 and 2019 in China. HRV parameters based on 24-h Holter and clinical risk factors associated with all-cause mortality were analyzed by multivariate Cox regression. The relationships between HRV and all-cause mortality were displayed by restricted cubic spline graphs. The predictive ability of nomogram model based on clinical risk factors and HRV were evaluated for survival rate.

**Results:** CKD5 patients included survival subgroup (n = 155) and all-cause mortality subgroup (n = 45), with the median follow-up time of 48 months. Logarithm of standard deviation of all sinus R-R intervals (lnSDNN) (4.40 ± 0.39 *vs*. 4.32 ± 0.42; *p* = 0.007) and logarithm of standard deviation of average NN intervals for each 5 min (lnSDANN) (4.27 ± 0.41 *vs*. 4.17 ± 0.41; *p* = 0.008) were significantly higher in survival subgroup than all-cause mortality subgroup. On the basis of multivariate Cox regression analysis, the lnSDNN (HR = 0.35, 95%CI: 0.17–0.73, *p* = 0.01) and lnSDANN (HR = 0.36, 95% CI: 0.17–0.77, *p* = 0.01) were associated with all-cause mortality, their relationships were negative linear. Spearman’s correlation analysis showed that lnSDNN and lnSDANN were highly correlated, so we chose lnSDNN, sex, age, BMI, diabetic mellitus (DM), β-receptor blocker, blood glucose, phosphorus and ln intact parathyroid hormone (iPTH) levels to build the nomogram model. The area under the curve (AUC) values based on lnSDNN nomogram model for predicting 3-year and 5-year survival rates were 79.44% and 81.27%, respectively.

**Conclusion:** In CKD5 patients decreased SDNN and SDANN measured by HRV were related with their all-cause mortality, meanwhile, SDNN and SDANN were highly correlated. Nomogram model integrated SDNN and clinical risk factors are promising for evaluating their prognosis.

## Introduction

Chronic kidney disease (CKD) is a global disease with high incidence and brings heavy social and economic burden. The United States Renal Data System (USRDS, 2021) reported that 14.90% of the U.S. adult population were diagnosed as CKD, which was associated with increased risks of cardiovascular diseases (CVD) and case fatality (“USRDS,” 2021), (Tonelli, et al., 2018). Traditional risk factors associated with CVD include hypertension, smoking, diabetic mellitus (DM) and anemia, etc (Deli, et al., 2013). Furthermore, the important role of abnormal heart rate circadian rhythm in CVD and all-cause mortality have been revealed in patients suffering from myocardial infarction or type 2 DM (La Rovere, et al., 1998; Bosone, et al., 2017).

Heart rate variability (HRV) parameters reflect the circadian rhythm of heart rate. Recorded by a non-invasive, repeatable and convenient clinical 24-h Holter examination, HRV parameters include SD of the normal-to-normal R-R intervals (SDNN), SD of 5-min average of normal R-R intervals (SDANN), root mean square of differences between adjacent normal R-R intervals (rMSSD), proportion of adjacent R-R intervals differing by>50 ms over 24 h (pNN50%), very low frequency (VLF), low frequency (LF), high frequency (HF), LF/HF ratio, et al., which are regulated by sympathetic and parasympathetic activities (Chou, et al., 2016). The frequency domain analysis showed that reduced HRV are associated with poorly controlled type 2 DM (Nganou-Gnindjio, et al., 2018). Five-minute HRV, particularly LF, are suggested to be promising risk predictors for coronary heart disease (Kotecha, et al., 2012). Compared with healthy controls, HRV parameters such as SDNN, SDANN, pNN50%, LF/HF, LF are lower in CKD patients (Cui, et al., 2021; J.; Zhang, et al., 2013), and related with their all-cause mortality and long-term prognosis (Mylonopoulou, et al., 2010; Chandra, et al., 2012; Kouidi, et al., 2013; Chang, et al., 2020). However, which HRV parameters are associated with all-cause mortality in CKD are controversial. Whether CKD related clinical risk factors combined with HRV can improve the predictive ability for their death remains uncertain.

Nomogram is a number of rulers where variables are listed separately, with a number of points assigned to a given magnitude of the variable. The scores obtained by the sum of all variables are matched to a scale of outcome (Balachandran, et al., 2015). Nomogram is used widely in clinical practice, especially in Oncology and medical outcomes, can make individualized estimates of prognosis for specific patients. In CKD patients, relevant clinical prognostic factors include body mass index (BMI), DM, mineral and bone metabolism disorders and medication history, etc. However, there are currently no study including above factors with HRV to investigate the predictive value for all-cause mortality in CKD patients.

Taking advantage of well-established CKD5 study cohort that initiated at Nanjing China, we screened HRV parameters which were correlated with all-cause mortality. Furthermore, their predictive value combined with clinical risk factors was analyzed by nomogram model in order to facilitate clinical utility.

## Materials and Methods

### Study Population

In this retrospective cohort study, 231 CKD5 patients were enrolled from the First Affiliated Hospital of Nanjing Medical University and the Affiliated BenQ Hospital of Nanjing Medical University in China from March 2011 to December 2019.

The enrolled patients were aged 18–75 years and had an estimated glomerular filtration rate (CKD-EPI equation) < 15 ml/min/1.73 m^2^. The dry weight of the dialysis patients was stable for at least 1 month to avoid the effects of overhydration on heart rate rhythm. The exclusion criteria were as follows: 1) undergoing maintenance dialysis between 0 and 3 months; 2) history of parathyroidectomy (PTX); 3) history of kidney transplantation; 4) fasting blood glucose on the day of evaluation≥200 mg/dl; 5) presence of fever, infection, pregnancy or lactating women; 6) severe congenital heart disease, atrial fibrillation or flutter, high-grade atrioventricular block, or permanent pacemaker implantation; 7) severe hepatic disease, chronic obstructive lung disease, malignant tumors, or severe mental disorders; 8) episodes of acute myocardial infarction, stroke, or a major surgical procedure within the past 2 months; 9) use of immunosuppressive medications, calcitonin, or bisphosphonates.

### Data Collection

The baseline characteristics of the patients were collected as follows: demographic information, dialysis mode, co-morbidities, causes of end stage kidney disease (ESKD) and history of CKD treatment. The primary end point was all-cause mortality. We followed up the patients by a phone interview or reviewing their medical records up to August 2019. The primary outcome was censored on the date of the last follow-up, and the patients who did not experience the primary outcome had their survival times censored. A further 31 (13.42%) participants who were uncontactable were not followed up.

### Measurements of Blood Parameters

Venous blood samples were tested in the morning after overnight fasting. For hemodialysis patients, blood samples were tested before dialysis. Routine blood tests were performed using an LH 750 Hematology Analyzer (Beckman Coulter). Biochemical indicators were measured by an automated biochemical analyzer (AU5400; Olympus Corporation). Serum intact parathyroid hormone (iPTH) levels were measured with second-generation iPTH assay kits using a UniCel DxI 800 Access Immunoassay System (Beckman Coulter).

### HRV Measurements

Each patient underwent 24-h Holter electrocardiogram (MARS Ambulatory ECG Analysis System; GE Healthcare). For hemodialysis patients, the dynamic electrocardiogram was analyzed on the nondialysis days.

HRV can be quantified using time-domain analysis and frequency-domain analysis, time-domain analysis consisting of MEANNN, SDNN, SDANN, rMSSD, pNN50%. Frequency-domain analysis consists of HF, VLF, LF, LF/HF.

### Statistical Analysis

Normally distributed data were described as mean ± standard deviation, the *t*-test and non-parametric test were used to compare continuous variables between groups. Categorized variables were expressed as frequency and analyzed by Chi-square test. Baseline characteristics of preterm cases and controls were compared using the *t*-test or Mann-Whitney *U* test for continuous variables depending on the data distribution, and chi-square test was used for categorical variables. Baseline factors that were differentially distributed in the survival and dead groups, including age, sex, BMI, DM, ln alkaline phosphatase (lnALP), lniPTH, phosphorus, β-receptor blocker were considered covariates of the statistical models. Log-transformed *t*-test was performed to compare HRV parameters between survival and dead group. ALP, iPTH and HRV risk factors were log_e_ transformed before statistical analysis as they had left-skewed distributions.

Multivariable Cox hazard proportional regression was performed to evaluate the associations between HRV parameters and all-cause mortality. To further explore nonlinear relationships between the base level of lnSDNN, lnSDANN and risk of all-cause mortality, restricted cubic spline^10^ was used with four knots at the 20th, 40th, 60th, and 80th percentiles. The 10th percentile of the predictor was the reference in corresponding curve by the R package of “rms” along with “ggplot2”. We also applied the multivariable Cox regression to predict the all-cause mortality using the HRV parameters. Spearman’s correlation analyzed the relationships between lnSDNN and lnSDANN. A nomogram for predicting all-cause mortality of these patients was developed on the basis of multivariable Cox regression analysis results. The sensitivity and specificity in predicting the long-term prognosis of CKD patients, were evaluated by the area under the curve (AUC) value of the receiver operating characteristic (ROC) curve by using the “timeROC” package in R.

All the statistical analyses were performed using R Software Version 3.6.2 (The R Foundation for Statistical Computing), and two-sided *p* < 0.05 was considered statistically significant unless stated otherwise.

### Ethics Statement

This study was approved by the Research Ethics Committee of the First Affiliated Hospital of Nanjing Medical University, Nanjing, China (ethics approval numbers: 2011-SR-072 and 2019-SR-368). All participants provided the informed consent. This research was designed in accordance with the Declaration of Helsinki.

## Results

### Baseline Characteristics of Participants

**FIGURE 1:**
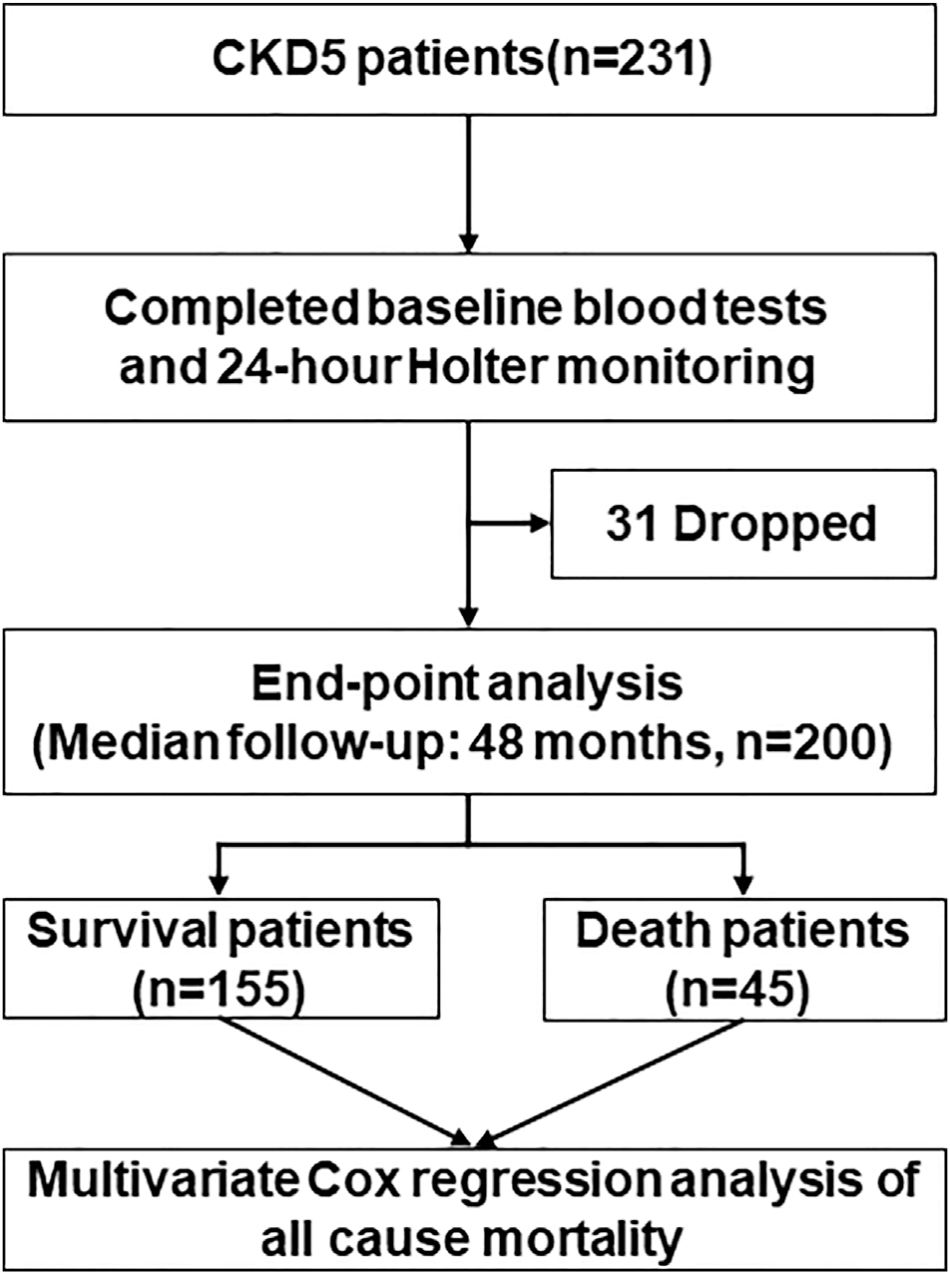
Flow diagram of the study.

A total of 200 CKD5 patients were divided into two subgroups: 45 patients with all-cause mortality and 155 patients with survival. Baseline data, hematology and HRV examinations were completed (Figure 1). There were 90 women and 110 men enrolled in our study, the average age was 52.26 ± 13.25 years old and median dialysis vintage was 12.0 months. Compared with survival group, the patients with all-cause mortality had older age and lower blood glucose levels, furthermore, their dialysis vintage was longer. The all-cause mortality group had more patients with diabetic nephropathy and fewer patients with chronic glomerulonephritis (CGN). The baseline demographics, clinical characteristics and laboratory results in survival and all-cause mortality subgroup of CKD5 patients are summarized in Table 1.

**TABLE 1:** Baseline demographics, clinical characteristics and laboratory results in survival and all-cause mortality subgroups of CKD5 patients.

Clinical characteristics	Overall (n = 200)	Survival subgroup (n = 155)	All-Cause Mortality subgroup (n = 45)	*p*-Value
**Demographics**				
Female/Male	90/110 (45)	70 (45.16)	20 (44.44)	1.000
Age (years)	52.26 ± 13.25	49.85 ± 13.13	61 ± 9.98	<0.001
BMI (kg/m^2^)	22.36 ± 3.43	22.56 ± 3.52	21.22 ± 3.06	0.125
Systolic BP (mmHg)	148.98 ± 25.67	148.45 ± 24.60	145 ± 29.29	0.590
Diastolic BP (mmHg)	85.62 ± 13.46	85.96 ± 14.06	81 ± 11.20	0.507
Dialysis mode, n (%)				
Predialysis	53 (26.50)	47 (30.32)	6 (13.33)	0.037
Hemodialysis	121 (60.50)	89 (57.42)	32 (71.11)	0.138
Peritoneal dialysis	27 (13.50)	20 (12.90)	7 (15.56)	0.833
Dialysis vintage(months)	12 (0-40.25)	9 (0-36)	24 (8-54)	0.006
**Comorbidities, n (%)**				
Diabetic mellitus	41 (20.50)	26 (16.77)	15 (33.33)	0.027
Hypertension	161 (80.50)	126 (81.29)	35 (77.78)	0.757
**Cause of ESKD, n (%)**				
Glomerulonephritis	132 (66)	109 (70.32)	23 (51.11)	0.027
Diabetic nephropathy	31 (15.50)	18 (11.61)	13 (28.89)	0.010
Hypertensive nephropathy	23 (11.50)	20 (12.90)	3 (6.67)	0.374
Polycystic kidney disease	14 (7)	13 (8.39)	1 (2.22)	0.274
Others	19 (9.50)	11 (7.10)	8 (17.78)	0.063
**Medication history, n (%)**				
Dihydropyridine CCBs	132 (66)	104 (67.10)	28 (62.22)	0.668
ACEI/ARB	44 (22)	33 (21.29)	11 (24.44)	0.806
β-receptor blocker	67 (33.50)	53 (34.19)	14 (31.11)	0.837
Phosphate binders	62 (43.36)	53 (43.80)	9 (40.91)	0.986
Active vitamin D sterols	59 (41.26)	53 (43.80)	6 (27.27)	0.225
Cinacalcet	8 (5.59)	8 (6.61)	0	0.461
**Laboratory values**				
Hemoglobin (g/L)	96.18 ± 22.39	96.67 ± 23.17	96 ± 19.59	0.566
Glucose (mmol/L)	5.44 ± 2.25	5.25 ± 1.84	4.88 ± 3.23	0.024
Creatinine (μmol/L)	868.35 ± 351.42	890.07 ± 363.04	747.70 ± 299.73	0.105
Urea (mmol/L)	25.51 ± 9.51	26.11 ± 9.60	21.95 ± 9.02	0.102
Total cholesterol (mmol/L)	4.44 ± 1.26	4.37 ± 1.21	4.41 ± 1.39	0.135
Triglyceride (mmol/L)	1.66 ± 1.03	1.61 ± 0.94	1.41 ± 1.27	0.223
Albumin (g/L)	37.50 ± 5.79	37.78 ± 5.52	38 ± 6.62	0.193
Calcium (mmol/L)	2.22 ± 0.28	2.21 ± 0.26	2.23 ± 0.34	0.608
Adjust Calcium (mmol/L)	2.27 ± 0.26	2.26 ± 0.23	2.31 ± 0.35	0.264
Phosphorus (mmol/L)	1.92 ± 0.64	1.93 ± 0.61	1.77 ± 0.76	0.621
ALP(U/L)	81.80 (63.62-109.22)	78.70 (61.45-106.00)	92.20 (77.10-113.10)	0.549
ln [ALP] (U/L)	4.48 ± 0.51	4.45 ± 0.54	4.52 ± 0.41	0.060
iPTH (pg/ml)	198.85 (96.13-470.70)	193.70 (88.40-468.25)	219.10 (148-479)	0.963
ln [iPTH] (pg/ml)	5.24 ± 1.27	5.23 ± 1.24	5.39 ± 1.36	0.821

Abbreviations: CKD, chronic kidney disease; BMI, body mass index; BP, blood pressure; ESKD, end-stage kidney disease; CCB, calcium channel blocker; ACEI/ARB, angiotensin-converting enzyme inhibitors/angiotensin receptor blocker; ALP, alkaline phosphatase; iPTH, intact parathyroid hormone; ln, the natural logarithm. Data are presented as mean ± SD, numbers and percentages, as appropriate. Other causes of ESKD, included obstructive nephropathy, interstitial nephritis, renovascular disease, lupus nephritis, and some unknown causes.

### Baseline HRV Parameters in CKD5 Patients

Compared with all-cause mortality subgroup, SDNN [82 (67-112) *vs*. 75 (52-84); *p =* 0.010] and SDANN [71 (56-97.50) *vs*. 65 (45-76); *p* = 0.006] were significantly higher in survival patients. LnSDNN (4.40 ± 0.39 *vs*. 4.32 ± 0.42; *p* = 0.007) and lnSDANN (4.27 ± 0.41 *vs*. 4.17 ± 0.41; *p* = 0.008) were also higher in survival group than all-cause mortality group. There was no significant difference between two subgroups in other time-domain and all of frequency-domain parameters (Table 2).

**TABLE 2:** Baseline HRV indexes in survival and all-cause mortality subgroups of CKD5 patients.

Heart rate variability	Overall (n = 200)	Survival Subgroup (n = 155)	All-Cause mortality Subgroup (n = 45)	*p*-Value
MHR (bpm)	80.06 ± 11.98	80.08 ± 11.87	79 ± 12.47	0.970
**Time domain measures**				
MEANNN (ms)	763 (684-835)	757.50 (680.75-835)	781 (704-848)	0.237
SDNN (ms)	80 (62.25-106)	82 (67-112)	75 (52-84)	0.010
SDANN (ms)	70 (54-91)	71 (56-97.50)	65 (45-76)	0.006
rMSSD (ms)	17 (13-23)	18 (14-23)	15 (12-23)	0.467
PNN50(%)	3.85 ± 6.42	3.64 ± 5.80	1.50 ± 8.24	0.389
lnMEANNN (ms)	6.63 ± 0.15	6.63 ± 0.15	6.66 ± 0.16	0.266
lnSDNN (ms)	4.36 ± 0.40	4.40 ± 0.39	4.32 ± 0.42	0.007
lnSDANN(ms)	4.23 ± 0.42	4.27 ± 0.41	4.17 ± 0.41	0.008
lnrMSSD (ms)	2.90 ± 0.46	2.90 ± 0.44	2.71 ± 0.56	0.915
**Frequency domain measures**				
VLF	96.86 (18.37-321.80)	30.30 (17.40-332.50)	134.38 (46.75-292.67)	0.217
LF	14.02 (8.34-55.76)	13.04 (8.98-53.61)	19.28 (7.02-62.95)	0.153
HF	7.98 (4.50-27.51)	7.84 (4.62-24.67)	14.36 (3-29.79)	0.188
LF/HF	1.75 (1.27-3.90)	1.75 (1.31-2.94)	1.91 (1-5.45)	0.969
lnVLF	4.42 ± 1.66	4.31 ± 1.71	4.90 ± 1.47	0.078
lnLF	3.11 ± 1.42	3.07 ± 1.38	2.96 ± 1.57	0.529
lnHF	2.22 ± 1.76	2.20 ± 1.65	2.66 ± 2.10	0.760
lnLF/HF	0.88 ± 1.08	0.87 ± 1.04	0.65 ± 1.19	0.758

Abbreviations: HRV, heart rate variability; MHR, mean heart rate; MEANNN, mean normal-to-normal R–R intervals; SDNN, SD of normal-to-normal R–R intervals; SDANN, SD of 5-minute average of normal R–R intervals; rMSSD, root mean square of differences between adjacent normal R–R intervals; pNN50%, proportion of adjacent R–R intervals differing by 50 ms over 24 h; VLF, very low frequency; LF, low frequency; HF, high frequency. ln, the natural logarithm based on e. Data are presented as mean ± SD, numbers and percentages, as appropriate.

### Associations between Clinical Characteristics and Risks for All-Cause Mortality in CKD5 Patients

After putting all clinical characteristics data in Supplementary Table S1 into the multivariate Cox regression model, we found that age (HR = 1.09, 95%CI:1.06–1.11, *p* < 0.001), DM (HR = 3.63, 95%CI:2.05–6.42, *p* < 0.001), β-receptor blocker (HR = 0.54, 95%CI:0.31–0.95, *p* = 0.032), blood glucose levels (HR = 0.54, 95%CI:0.31–0.95, *p* = 0.032), blood phosphorus levels (HR = 0.58, 95%CI:0.36–0.93, *p* = 0.024), and lniPTH (HR = 0.74, 95%CI:0.63–0.86, *p* < 0.001) were demonstrated as independent predictors for all-cause mortality in CKD5 patients. Sex (HR = 0.97, 95%CI: 0.59–1.62, *p* = 0.920) and BMI (HR = 0.94, 95%CI:0.87–1.02, *p* = 0.118) were not associated with all-cause mortality (Table 3).

**TABLE 3:** Multivariate cox regression of clinical characteristics for all-cause mortality in CKD5 patients.

Clinical characteristics	HR (95%CI)	*p*-Value
**Demographics**		
Female/Male	0.97 (0.59,1.62)	0.920
Age (years)	1.09 (1.06,1.11)	<0.001
BMI (kg/m^2^)	0.94 (0.87,1.02)	0.118
Systolic BP (mmHg)	1.00 (0.99,1.01)	0.448
Diastolic BP (mmHg)	0.98 (0.96,1.00)	0.016
**Dialysis mode, n (%)**		
Predialysis	0.98 (0.42,2.28)	0.969
Hemodialysis	0.73 (0.41,1.31)	0.288
Peritoneal dialysis	1.63 (0.80,3.30)	0.178
Dialysis vintage(months)	1.00 (0.99,1.00)	0.264
**Comorbidities, n (%)**		
Diabetic mellitus	3.63 (2.05,6.42)	<0.001
Hypertension	0.86 (0.49,1.52)	0.606
**Cause of ESKD, n (%)**		
Glomerulonephritis	0.38 (0.23,0.63)	0.000
Diabetic nephropathy	4.24 (2.29,7.82)	<0.001
Hypertensive nephropathy	0.37 (0.05,2.65)	0.320
Polycystic kidney disease	0.62 (0.15,2.55)	0.512
Others	1.86 (0.89,3.92)	0.100
**Medication history, n (%)**		
Dihydropyridine CCBs	0.95 (0.57,1.57)	0.838
ACEI/ARB	0.79 (0.42,1.46)	0.456
β-receptor blocker	0.54 (0.31,0.95)	0.032
Phosphate binders	0.76 (0.38,1.53)	0.442
Active vitamin D sterols	0.87 (0.43,1.78)	0.708
Cinacalcet	0.32 (0.08,1.33)	0.118
**Laboratory values**		
Hemoglobin (g/L)	0.99 (0.98,1.00)	0.060
Glucose (mmol/L)	1.20 (1.11,1.31)	<0.001
Creatinine (μmol/L)	0.9986 (0.9977,0.9996)	0.004
Urea (mmol/L)	0.99 (0.96,1.03)	0.688
Total cholesterol (mmol/L)	1.20 (0.97,1.48)	0.091
Triglyceride (mmol/L)	1.00 (0.84,1.20)	0.967
Albumin (g/L)	0.91 (0.87,0.96)	0.000
Adjust calcium (mmol/L)	0.48 (0.20,1.18)	0.110
Phosphorus (mmol/L)	0.58 (0.36,0.93)	0.024
ln [ALP] (U/L)	0.9987 (0.9977,0.9998)	0.019
ln [iPTH] (pg/ml)	0.74 (0.63,0.86)	<0.001

Abbreviations: CKD, chronic kidney disease; BMI, body mass index; BP, blood pressure; ESKD, end-stage kidney disease; CCB, calcium channel blocker; ACEI/ARB, angiotensin-converting enzyme inhibitors/angiotensin receptor blocker; ALP, alkaline phosphatase; iPTH, intact parathyroid hormone; ln, the natural logarithm. Other causes of ESKD included obstructive nephropathy, interstitial nephritis, renovascular disease, lupus nephritis, and some unknown causes. HR, hazard ratio; CI, confidence interva.

**FIGURE 2:**
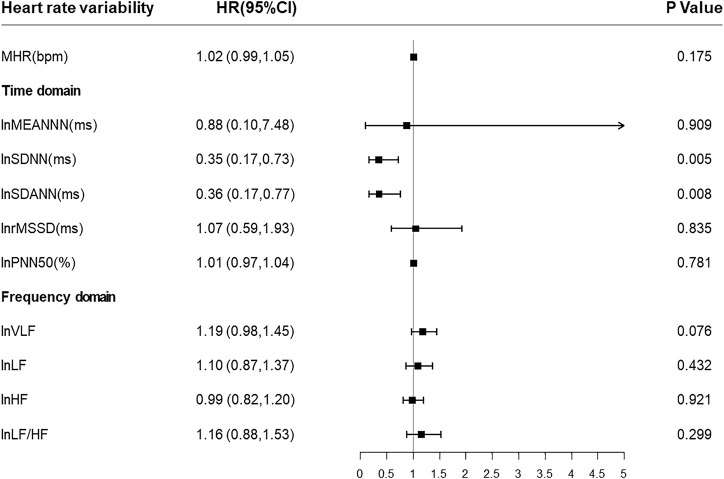
Forest plots of associations between HRV parameters and all-cause mortality. Present the estimates with a horizontal line representing 95% confidence intervals (CIs). Abbreviations: HRV, heart rate variability; SDNN, SD of normal-to-normal R-R intervals; SDANN, SD of 5-minute average of normal R-R intervals.

Then we put all HRV parameters to determine the independent predictors for all-cause mortality in CKD5 patients. Cox regression analysis revealed that lnSDNN (HR = 0.35, 95% CI:0.17–0.73, *p* = 0.005) and lnSDANN (HR = 0.36, 95% CI:0.17–0.77, *p* = 0.008) are independent risk factors for all-cause mortality with adjustment for age, sex (male, 0; female, 1), BMI, diabetic mellitus (yes or no), ln (iPTH), phosphorus and drug intaking of β-receptor blocker (Figure 2).

### Non-Linear Relationships between LnSDNN/LnSDANN and All-Cause Mortality in CKD5 Patients

**FIGURE 3:**
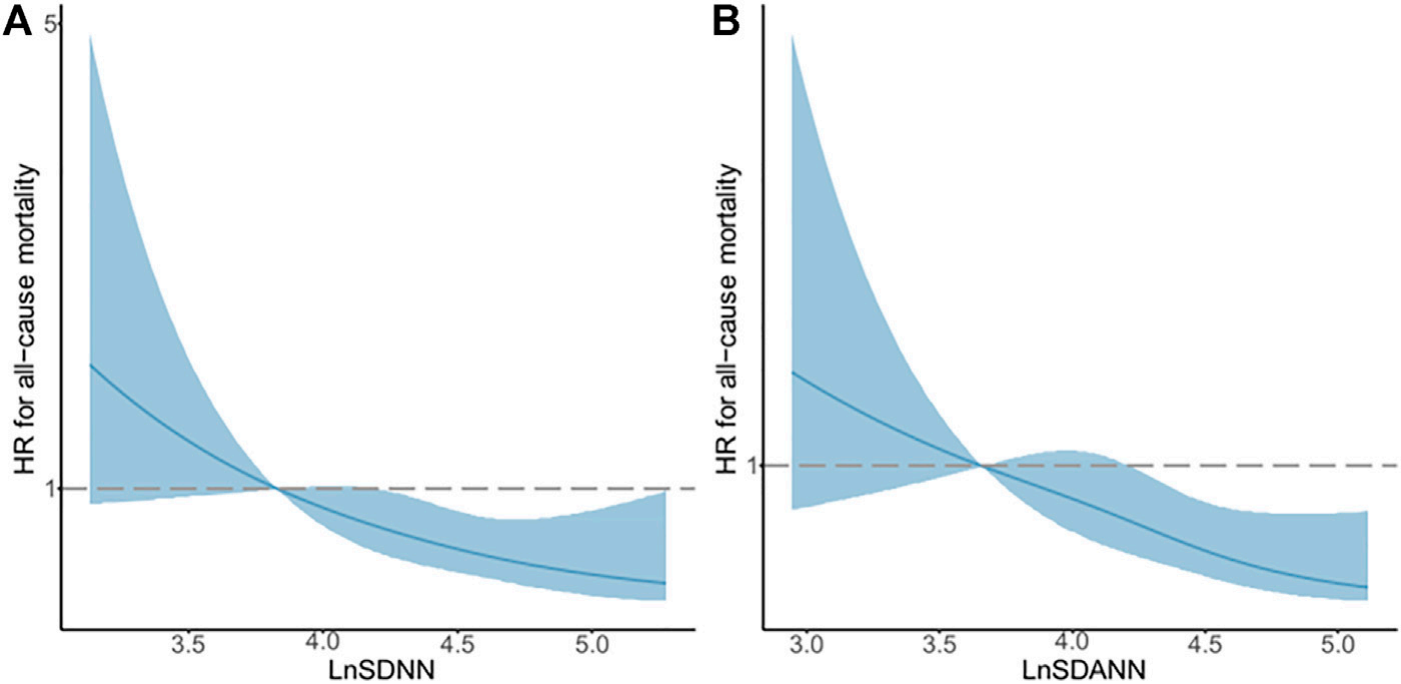
Associations between LnSDNN/LnSDANN and All-cause Mortality Displayed by Restricted Cubic Spline Analysis in CKD5 Patients. **(A)** lnSDNN and all-cause mortality. **(B)** lnSDANN and all-cause mortality. Hazard ratios are indicated by solid blue lines and 95% confidence intervals by shaded areas. Reference lines for no association are indicated by dashed lines at a hazard ratio of 1.0. Abbreviations: SDNN, SD of normal-to-normal R-R intervals; SDANN, SD of 5-minute average of normal R-R intervals. CKD, chronic kidney disease.

**FIGURE 4:**
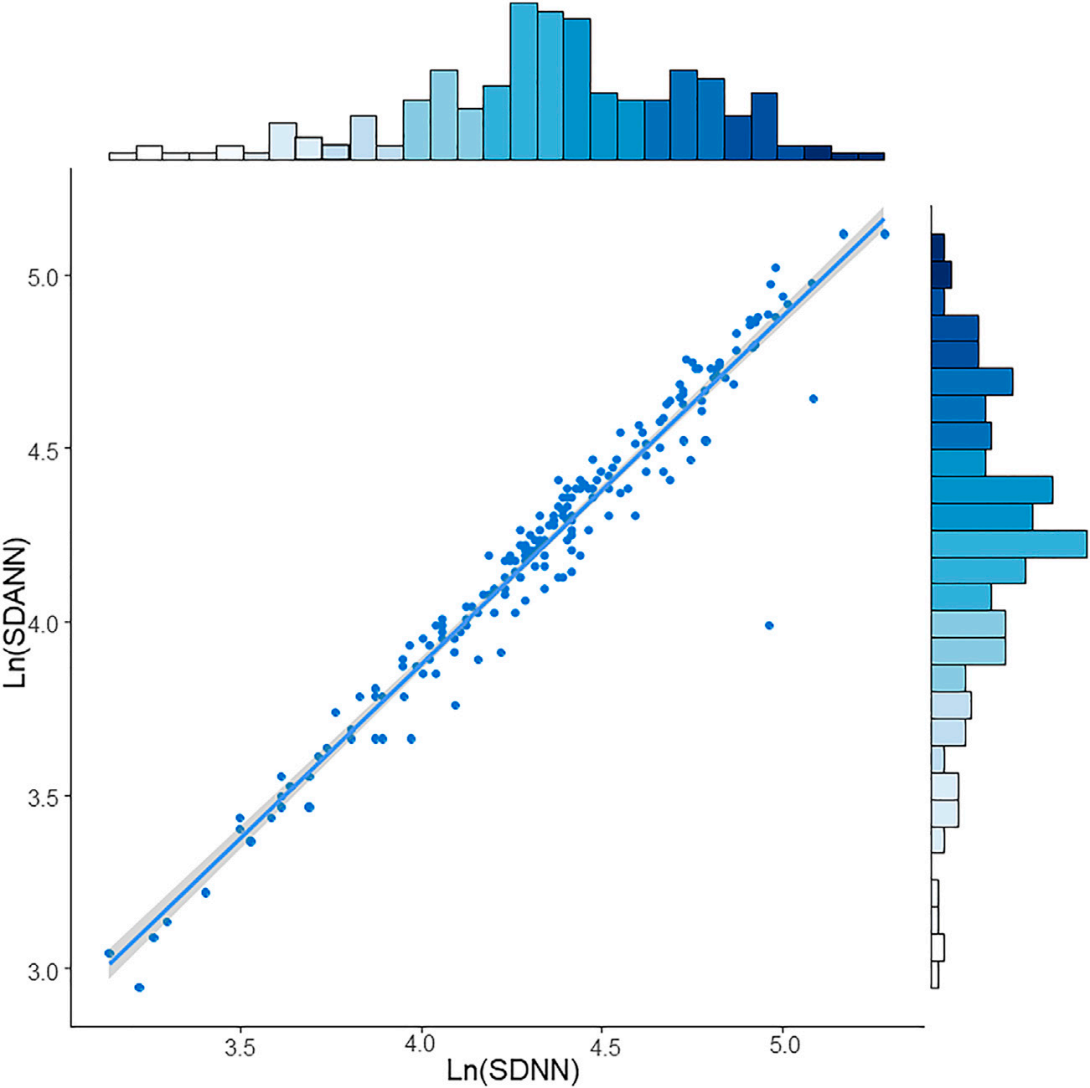
Spearman’s correlation between LnSDNN and LnSDANN in CKD5 patients. Abbreviations: SDNN, SD of normal-to-normal R-R intervals; SDANN, SD of 5-minute average of normal R-R intervals. CKD, chronic kidney disease.

The restricted cubic spline regression analysis between lnSDNN/lnSDANN and the risks of all-cause mortality in CKD5 patients was established (Figure 3). There was a negative correlation between lnSDNN and the risk of all-cause mortality (*p* for non-linearity = 0.452), the same outcome had been found between lnSDANN and the risk of all-cause mortality (*p* for non-linearity = 0.899) in 200 CKD5 patients. Restricted cubic spline analysis revealed linear relationships between lnSDNN/lnSDANN and all-cause mortality. Indicating that as the increase of lnSDNN and lnSDANN, all-cause mortality of CKD5 patients decreased. Spearman’s correlation analysis proved that lnSDNN and lnSDANN were highly correlated (r = 0.97, *p* < 0.001) (Figure 4).

### Nomogram Model Based on Clinical Risk Factors and LnSDNN for Predicting All-Cause Mortality in CKD5 Patients

Multivariate Cox regression analysis proved that the risk factors for all-cause mortality in CKD5 patients included age, DM, β-receptor blocker, blood glucose, phosphorus and lniPTH levels. Spearman’s correlation analysis indicated that lnSDNN and lnSDANN were highly correlated, thus lnSDNN, above clinical factors, sex and BMI were implemented by nomogram model.

**FIGURE 5:**
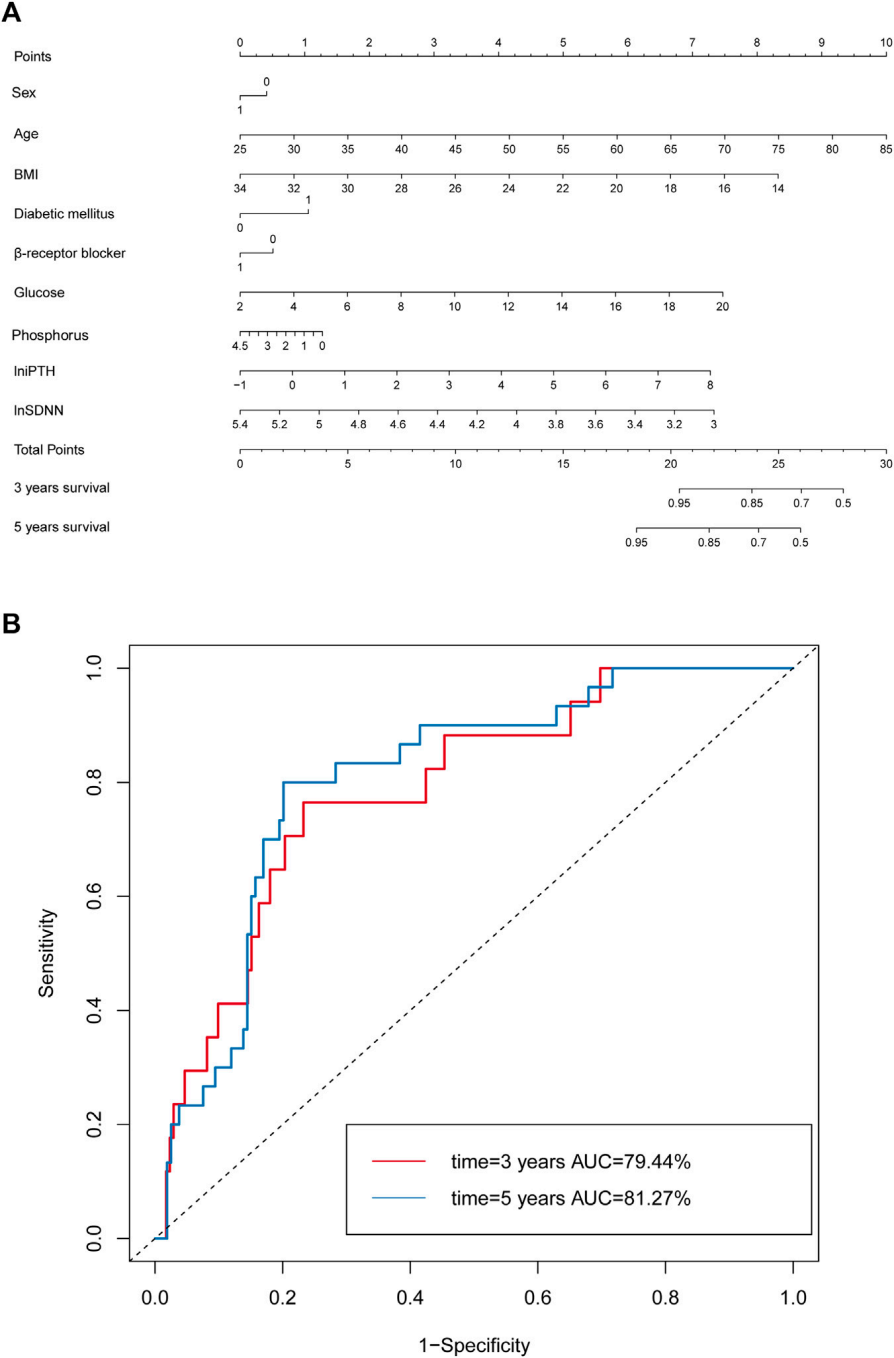
Nomogram model based on clinical risk factors and HRV to predict all-cause mortality in CKD5 patients. **(A)** Nomogram model based on clinical risk factors and lnSDNN for all-cause mortality. **(B)** ROC validating the discriminatory power of the nomogram model for 3-year and 5-year survival rates in CKD5 patients. Abbreviations: HRV, heart rate variability; SDNN, SD of normal-to-normal R-R intervals; CKD, chronic kidney disease; ROC, receiver operator characteristic curve; AUC, area under ROC.

The prediction performance of the nomogram was further confirmed by AUC analysis. The AUC of lnSDNN for 3-year mortality was 0.79 (95% CI: 0.69-0.90) with sensitivity of 88.24% and specificity of 53.93%, and for 5-year mortality was 0.81 (95% CI: 0.74-0.89) with sensitivity of 90.00% and specificity of 57.58%, respectively. These results demonstrated that the nomogram based on clinical risk factors and lnSDNN had a great discriminatory ability in predicting all-cause mortality for CKD5 patients. Moreover, AUC showed good predictive power for both 3-year and 5-year mortality (Figure 5A,B,).

## Discussion

Appropriate screening, classification and management for CKD5 patients are of great importance to prevent poor prognosis of CKD, including CVD and mortality. It has been suggested that autonomic dysfunctions indicated by lower HRV are associated with higher all-cause mortality and CVD, especially in patients with advanced CKD (Chandra, et al., 2012; “Heart rate variability: standards of measurement, physiological interpretation and clinical use. Task Force of the European Society of Cardiology and the North American Society of Pacing and Electrophysiology,” (Author Anonymous, 1996; Koomans, et al., 2004).

HRV parameters include Time-domain measures (Cygankiewicz, et al., 2013). SDNN, the SD of NN intervals, is the most common assessment. Both sympathetic nervous system (SNS) and parasympathetic nervous system (PNS) activities contribute to SDNN, which is highly correlated with Ultra-Low Frequency (ULF), VLF, LF band power, and total power (Umetani, et al., 1998). SDNN, recommended by the European Society of Cardiology (ESC) task force, is reported to be the “gold standard” for medical stratification of cardiac risk when recorded over a 24 h period (“Heart rate variability: standards of measurement, physiological interpretation and clinical use. Task Force of the European Society of Cardiology and the North American Society of Pacing and Electrophysiology,” 1996). Nolan revealed that SDNN was the powerful contributor for the risks of death in heart failure patients (Nolan, et al., 1998). When stratified analysis was performed, Agata found that SDNN <96 ms in CKD patients was an independent predictor for CVD with 2 years follow-up (Buonacera, et al., 2016). In hemodialysis patients, SDNN<75 ms was a strong predictor for all-cause mortality (Chou, et al., 2016).

SDANN, the SD of average NN intervals, is not a surrogate for SDNN since it is calculated using 5 min segments instead of an entire 24 h series (Kuusela, 2012). European Society of Cardiology and the North American Society of Pacing and Electrophysiology (ESC/NASPE) Task Force on HRV analysis illustrated that decreased SDANN was verified to predict both all-cause mortality and CVD (“Heart rate variability: standards of measurement, physiological interpretation and clinical use. Task Force of the European Society of Cardiology and the North American Society of Pacing and Electrophysiology,” 1996). However, Shaffer F reported that SDANN did not provide additional meaningful information (Shaffer, et al., 2014). Letian Yang confirmed that decreased SDANN and LF/HF were associated with all-cause death and CVD, and decreased SDNN was the only identified predictor for CVD in maintenance hemodialysis patients (Yang, et al., 2021). In non-diabetic HD patients, lower SDNN or SDANN group developed more major adverse cardiac and cerebrovascular event (MACCE) than higher groups (Kida, et al., 2017).

Here we confirmed that compared with survival subgroup, SDNN [75 (52-84) *vs*. 82 (67-112); *p =* 0.010] and SDANN [65 (45-76) *vs*. 71 (56-97.50); *p* = 0.006] were lower in all-cause mortality subgroup. Our team also reported that SDNN [82.7 ± 48.4 *vs*. 140.9 ± 34.9); *p <* 0.001] and SDANN [74.7 ± 61.5 *vs*. 128.4 ± 34.2; *p* < 0.001] in CKD5 patients were significantly lower when compared with healthy populations (J. Zhang, et al., 2013).

In a recent study from Chinese population, Zhang et al. (2022) identified functional diurnal patterns and parameters by monitoring personalized, heart rate based diurnal changes. These findings have important implications for understanding how a regular heart diurnal pattern benefits cardiac function and raising the possibility of non-pharmacological intervention against circadian related CVD. Consistent with above and previous studies, we proved that both SDNN and SDANN were independent risk factors for all-cause mortality in CKD5 patients. Furthermore, we firstly used the restricted cubic spline regression models to show the negative linear relationships between them, with the increase of lnSDNN/lnSDANN and decrease of all-cause mortality in CKD5 patients.

There is a dearth of literature in the area of exact mechanisms of decreased HRV. Abnormal cardiac autonomic modulations are reported to be linked with increased serum phosphate levels and secondary hyperparathyroidism (SHPT), which contributes to a higher risk of sudden cardiac death (Poulikakos, et al., 2014). Our team found that successful PTX in severe SHPT patients may contribute to reverse the high CVD risks by blunting sympathetic hyperactivity and enhancing parasympathetic activity as indicated by normalized HRV parameters (J. Zhang, et al., 2013). Ng et al. (2017) reported that calcification of hand artery (HA) was associated with autonomic dysfunction, the patients with lower autonomic tone and severe HA calcification had the highest mortality rate. Fadaee et al. (2017) found that oxidative stress is significantly and independently associated with decreased HRV in patients with CKD.

The emerging research has been proved that the predictive power of HRV parameters was limited by low sensitivity (Nganou-Gnindjio, et al., 2018), and also influenced by variables such as age, sex, glucose, uremia toxin and drugs intake, etc (Kotecha, et al., 2012; Sarnak, et al., 2019). Among them β-receptor blocker has been reported to play an important role in improving HRV parameters (Mortara, et al., 2000; Kubo, et al., 2005).

Nomogram has been emerged as a simple tool with numerous advantages and estimated individualized risks based on the characteristics of patients and disease (Grimes, 2008). As a quantitative analysis tool, nomogram overcomes the shortcomings of hierarchical analysis. In order to improve the predictive value of HRV parameters and considering various clinical related factors, we firstly applied nomogram constructed by sex, age, BMI, DM, β-receptor blocker, glucose, phosphorus, lniPTH, lnSDNN to predict the risks of all-cause mortality in CKD5 patients.

To our knowledge, there is no study demonstrated a nomogram based on HRV to predict all-cause mortality for CKD5 patients. We developed simple and easy-to-use prognostic model integrating clinical parameters for CKD5 patients from the perspective of heart rate circadian rhythm, and the nomogram model is promising for evaluating their all-cause mortality. Moreover, we discovered that there is collinearity between SDANN and SDNN. This finding is consistent with Shaffer F’s study which confirmed that SDANN did not provide additional useful information than SDNN (Shaffer, et al., 2014).

Our research has several potential limitations. The lack of external validation studies, the rather long recruitment period and a low rate of in-hospital limited our study by reducing the power to detect possible associations between HRV and all-cause mortality for CKD5 patients. In the future, we will continue to incorporate more data of CKD5 patients, and conduct external validation of nomograms to confirm its sensitivity and specificity, so as to facilitate clinical application.

## Conclusion

Here we proved that decreased heart rate variability parameters SDNN and SDANN were negatively correlated with all-cause mortality in CKD5 patients. Spearman’s analysis showed that lnSDNN and lnSDANN were highly correlated. Based on SDNN and clinical risk factors, we established the first practical nomogram that can predict individualized prognosis of CKD5 patients from the perspective of heart rate circadian rhythm, the nomogram is promising for evaluating their all-cause mortality with high accuracy and reliability. Our study may provide new insight for improved decisions and treatments of CKD patients, in order to decrease their all-cause mortality.

## Data Availability

The raw data supporting the conclusion of this article will be made available by the authors, without undue reservation.
