# Supplementary material for: Nomogram Model Based on Clinical Risk Factors and Heart Rate Variability for Predicting All-Cause Mortality in Stage 5 CKD Patients
Source: Front Genet. 2022 May 16;13:872920. doi: 10.3389/fgene.2022.872920 (PMC9149361; doi:10.3389/fgene.2022.872920)
Supplement: Supplementary file 1 [file Table1.DOCX]

**Supplemental Table 1. Multivariate Cox Regression Analysis of the Risk Factors for All-cause Mortality in CKD5 Patients**

| **Heart rate variability** | **Multivariate model**  **HR (95%CI)** | ***P*-value** |
| --- | --- | --- |
| **MHR(bpm)** | 1.019(0.992,1.048) | 0.175 |
| **Time domain** |  |  |
| lnMEANNN (ms) | 0.883 (0.104,7.478) | 0.909 |
| lnSDNN (ms) | 0.351 (0.169,0.729) | 0.005 |
| lnSDANN(ms) | 0.359 (0.168,0.765) | 0.008 |
| lnrMSSD (ms) | 1.065 (0.589,1.927) | 0.835 |
| lnPNN50(%) | 1.005(0.968,1.044) | 0.781 |
| **Frequency domain** |  |  |
| lnVLF | 1.192 (0.982,1.447) | 0.076 |
| lnLF | 1.095 (0.874,1.371) | 0.432 |
| lnHF | 0.991 (0.819,1.198) | 0.921 |
| lnLF/HF | 1.158 (0.878,1.526) | 0.299 |

**Abbreviations:** HR, Hazard Ratio; CI, confidence interval; MHR, mean heart rate; MEANNN, mean normal-to-normal R–R intervals; SDNN, SD of normal-to-normal R–R intervals; SDANN, SD of 5-minute average of normal R–R intervals; rMSSD, root mean square of differences between adjacent normal R–R intervals; pNN50%, proportion of adjacent R–R intervals differing by 50 ms over 24 hours; VLF, very low frequency; LF, low frequency; HF, high frequency.
